# Impact of residual urine volume decline on the survival of chronic hemodialysis patients in Kinshasa

**DOI:** 10.1186/s12882-016-0401-9

**Published:** 2016-11-21

**Authors:** Vieux Momeme Mokoli, Ernest Kiswaya Sumaili, François Bompeka Lepira, Jean Robert Rissassy Makulo, Justine Busanga Bukabau, Patrick Parmba osa Izeidi, Jeannine Losa Luse, Stéphane Kalambay Mukendi, Désiré Kulimba Mashinda, Nazaire Mangani Nseka

**Affiliations:** 1 Division of Nephrology, University of Kinshasa, Kinshasa, Democratic Republic of the Congo; 2 Hemodialysis Unit of Ngaliema Medical Center, Kinshasa, Democratic Republic of the Congo; 3 Hemodialysis Unit of Provincial General Hospital of Kinshasa, Kinshasa, Democratic Republic of the Congo; 4 School of Public Health, University of Kinshasa, Kinshasa, Democratic Republic of the Congo

**Keywords:** Residual urine volume decline, Chronic hemodialysis, Survival, Kinshasa

## Abstract

**Background:**

Despite the multiple benefits of maintaining residual urine volume (RUV) in hemodialysis (HD), there is limited data from Sub-Saharan Africa. The aim of this study was to assess the impact of RUV decline on the survival of HD patients.

**Methods:**

In a retrospective cohort study, 250 consecutive chronic HD patients (mean age 52.5 years; 68.8% male, median HD duration 6 months) from two hospitals in the city of Kinshasa were studied, between January 2007 and July 2013. The primary outcome was lost RUV. Preserved or lost RUV was defined as decline RUV < 25 (median decline) or ≥ 25 ml/day/month, respectively. The second endpoint was survival (time-to death). Survival curves were built using the Kaplan-Meier methods. We used Log-rank test to compare survival curves. Predictors of mortality were assessed by Cox proportional hazards regression models.

**Results:**

The cumulative incidence of patients with RUV decline was 52, 4%. The median (IQR) decline in RUV was 25 (20.8–33.3) ml/day/month in the population studied, 56.7 (43.3–116.7) in patients deceased versus 12.9 (8.3–16.7) in survivor patients (*p* < 0.001). Overall mortality was 78 per 1000 patient years (17 per 1000 in preserved vs 61 per 1000 lost RUV). Forty six patients (18.4%) died from withdrawal of HD due to financial constraints. The Median survival was 17 months in the whole group while, a significant difference was shown between lost (10 months, *n* = 119) vs preserved RUV group (30 months, *n* = 131; *p* = 0001). Multivariate Cox proportional hazards models showed that, decreased RUV (adjusted HR 5.35, 95% CI [2.73–10.51], *p* < 0.001), financial status (aHR 2.23, [1.11–4.46], *p* = 0.024), hypervolemia (a HR 2.00, [1.17–3.40], *p* = 0.011), lacking ACEI (aHR 2.48, [1.40–4.40], *p* = 0.002) or beta blocker use (aHR 4.04, [1.42–11.54], *p* = 0.009), central venous catheter (aHR 6.26, [1.71–22.95], *p* = 0.006), serum albumin (aHR 0.93, [0.89–0.96], *p* < 0.001) and hemoglobin (aHR 0.73, [0.63–0.84], *p* < 0.001) had emerged as the independent predictors of all-cause mortality.

**Conclusion:**

More than half of HD patients in this cohort study experienced fast RUV decline which contributed substantially to increase mortality, highlighting the need for its prevention and management.

## Background

Despite advances in dialysis, mortality remains high, especially cardiovascular. According to the record of the 2012 Annual Report of the “European Renal Association-European Dialysis and Transplant Association, EDTA” an overall 5-year survival of patients treated by HD in 30 European countries was 59.7%, that is 39.3% for patients aged 65–74 years and 21.3% for those over 75 years old [1]. In addition, the 2013 American report of “United States Renal Data System, USRDS” stated a 5-year survival of 40% [2]. In Japan and Taiwan, a 5-year survival was estimated at 56.7% and 56% respectively [3, 4]. This high mortality in the developed countries has motivated the search and identification of potential predictors to help improving the survival of dialysis patients. This approach has the advantage of improving individual and collective acceptance of dialysis and rational use of the available resources, especially in resource-limited countries where any death of dialysis patient could lead to a reluctance of patients to accept this treatment.

Among the factors contributing to the improvement of patients’ survival on dialysis, residual renal function (RRF) or residual urine volume (RUV) appears to play a paramount role [5]. Indeed, several prospective, retrospective cohorts and observational studies have shown that preservation of RUV was independently associated with better survival [5–9] and that the expected benefit was beyond the one related to a better clearance of low molecular weight solutes [10]. In this regard, increasing evidence suggests that the preservation of RUV contribute in favorable way on the predictors of mortality in HD such as hypervolemia [11], left ventricular hypertrophy (LVH) and congestive heart failure [12, 13], heart rhythm disorders [14] and ischemic strokes [15]. In fact, Ma and Ding [16] found, in a case-control study, that the frequency of LVH and systolic dysfunction was significantly lower in patients with preserved RRF (RUV ≥ 200 ml/day) compared to those having lost RRF (RUV ˂ 200 ml/day). Furthermore, similar findings with respect to plasma concentration of B-type natriuretic peptide (BNP) and homocysteine were also reported [16].

Despite the multiple benefits of the preservation of RUV in patients on maintenance hemodialysis (HD), available data on the preservation of the RUV in sub-Saharan Africa are scarce. Hence, there is a need to fill this gap by acquiring reliable data that can inform the development of policy and rational and adapted strategies as regards extrarenal purification to better survival of chronic HD patients. The aim of this study was to investigate the impact of the RUV decline on mortality in our hemodialysis patients.

## Methods

In this retrospective cohort study, we selected all adults (≥18 years old) on maintenance at least 4 weeks of renal replacement therapy (RRT) for end-stage renal disease (ESRD) who began HD at two existing centers in city of Kinshasa from January 2nd, 2007 to July 31^st^ 2013.

### Data collection

#### Exposure

The initial RUV was directly measured at the hospital with a 24-h urine collection. It was assessed between two sessions of HD, at 3 months, 6 months, and 12 months after the beginning of HD. Information about urine volume was available for 250/250 (100%) participants at baseline, 200/212 (94.3%), 127/180 (70.5%) and 77/142 (54.2%) at 3, 6 and 12 months, respectively. Patients had a RUV preserved when its decline was lower than 25 ml/day/month (median of decline) between initiation of HD and death or at the end of the observation. Residual urine volume was considered as not preserved when its decline was greater than or equal to 25 ml/day/month.

#### Outcome

The primary outcome was lost RUV. The second endpoint was survival (time-to death). Patients were followed from the first reported date of dialysis to the date of death or July 31st, 2013, the end of the database period. Survival, mortality information and causes of death were obtained from clinic report and medical records of HD patients. Patients were divided into 2 groups according to survival between deceased and survivor. During this study, 38 participants died in the first 3 months, 33 others in the second quarter and 36 beyond the third quarter.

#### Other variables

The others parameters of interest were socio-demographic (age, gender, occupation, marital status, socioeconomic status, source of funding and education), clinical [height, weight, body mass index (BMI), delay between diagnosis of kidney disease and nephrology care or between indication and beginning of HD, primary renal disease, complications and comorbidities associated with ESRD on HD initiation, medical treatment throughout follow-up in dialysis and those specific to HD [type of vascular access, weekly time of HD, dialysis dose (Kt/V urea index)]. All these data were recorded on appropriate data collection form.

Funding was considered secured when financial support in HD was provided by one of the following facilities: company, government, foreign (mutual health organization, social security or health insurance). Systolic blood pressure (SBP) and diastolic blood pressure (DBP) were measured using an electronic sphygmomanometer before the session of HD, pulse pressure (PP) was defined as the difference between the SBP and DBP. Overweight and obesity were defined by a BMI ≥ 25 kg/m^2^ and ≥ 30 kg/m^2^ respectively [17]. Hypervolemia was defined as a central venous pressure ≥ 13 mm Hg at the initiation of HD [18].

The Charlson comorbidity index was used to categorize comorbidities as low, moderate, high and very high when the index was ≤ 3, 4–5, 6–7 and ≥ 8, respectively [19]. Inflammation was defined as a CRP level > 3 mg/l measured at 3 months of HD [20].

### Statistical analysis

All data were expressed as mean ± SD, median (interquartile range) or number (percentage), as appropriate. Statistical analyses were performed using SPSS version 21 for Windows (SPSS Inc, Chicago, IL) and Stata version 13. The Kaplan-Meier curves were built for survival analyses. Differences between survival curves based on the preservation or not of RUV were described using the Log-Rank test and the Chi-square test as appropriate. Survival was defined as the time period between the beginning of HD and the death or the end of the study. All patients deceased were considered uncensored. Censored patients were those alive (at the end of study), lost to follow up or those who were transplanted. Risk factors for mortality were assessed by performing univariate Cox regression analysis, and variables with *P value* < 0.05 were included in a multivariate analysis by applying a multiple Cox regression based on forward elimination of data. The significance level of *P* value was set at 0.05 or less on two-sided tests. Participants in this study have given their consent. The personal information had been identified and all the data were analyzed anonymously. This study was approved by the Ethics Committee of the Faculty of Medicine, University of Kinshasa (acceptance number ESP/CE/033/2015).

## Results

As shown in Table 1, 250 patients were included. At baseline, the patients on HD were 52.5 ± 12.3 years old, and 172 patients were male (69%). The average HD duration was 15 months. Excepted for SEL (socioeconomic level), funding of HD, several other variables were not different between deceased patients versus those who survived at the end of the study. Indeed, the survivors group had a higher socioeconomic level (*p* < 0.001) and a more secure funding HD in comparison to deceased patients (*p* < 0.001).

**Table 1:** Baseline Sociodemographic Characteristics of deceased and survivor patients

Variables	All Group (*N* = 250)	deceased (*N* = 107)	survivor (*N* = 143)	*p*
Gender, *n* (%)				0084
Male	172 (68.8)	71 (66.4)	101 (70.6)	
Female	78 (31.2)	36 (33.6)	42 (29.4)	
Mean age, years	52.5 ± 12.3	52.5 ± 13.2	52.4 ± 11.8	0.982
Marital Status, *n* (%)				0.602
Married	178 (71.2)	71 (66.4)	107 (74.8)	
Single	72 (28.8)	36 (33.6)	36 (25.2)	
Profession, *n* (%)				0.157
Unemployed	62 (24.8)	27 (25.2)	35 (24.5)	
Student	10 (4)	7 (6.5)	3 (2.1)	
Housewife	13 (5.2)	5 (4.7)	8 (5.6)	
Retired	19 (7.6)	12 (11.2)	7 (4.9)	
Worker	88 (35.2)	37 (34.6)	51 (35.7)	
Executive	58 (23.2)	19 (17.8)	39 (27.2)	
Education, *n* (%)				0.622
None	2 (0.8)	1 (0.9)	1 (0.7)	
Primary	24 (9.6)	11 (10.3)	13 (9.1)	
Secondary	88 (35.2)	43 (40.2)	45 (31.5)	
Superior	136 (54.4)	52 (48.6)	84 (58.7)	
SEL, *n* (%)				<0.001
Medium	204 (81.6)	99 (92.5)	105 (73.4)	
High	46 (18.4)	8 (7.5)	38 (26.6)	
Financing, *n* (%)				<0.001
Patient	49 (19.6)	19 (17.8)	30 (20.9)	
Family	98 (39.2)	59 (55.1)	39 (27.3)	
Firm	68 (27.2)	24 (22.4)	44 (30.8)	
State	14 (5.6)	3 (2.8)	11 (7.7)	
Insurance/Mutual Health Organization	21 (8.4)	2 (1.9)	19 (13.3)	
Delay diagnosis-nephrology care*(days), *n* = 215	29 (28–30)	30 (29–30)	27 (26–29)	0.922
Delay indication - start HD* (days), *n* = 231	28 (27–30)	29 (28–30)	27 (21–30)	0.239

Data are expressed as numbers and proportions in parentheses or mean ± standard deviation, median (interquartile range) as appropriate

Abbreviations: *SEL*: socio-economic level, *HD*: hemodialysis

*continuous (quantitative) variables

The cumulative incidence of patients with RUV decline was 52, 4%. The median (IQR) RUV for all the patients before starting HD, and at 3, 6 and 12 months were 550 (500–705), 500 (450–550), 500 (400–550) and 400 (200–530) ml/day, respectively. The median (IQR) decline in RUV was 25 ml/day/month (20.8–33.3) (Table 2). Compared to deceased patients, the survivors had higher median RUV regardless the time of observation: 840 vs 250 ml/day (*p* < 0.001) at initiation, 700 vs 260 ml/day (*p* < 0.001) after 3 months, 560 vs 250 ml/day (*p* < 0.001) after 6 months and 500 vs 200 ml/day (*p* = 0.003) after 12 months. In addition, a lower median decline [12.9 (8.3–16.7)] was encountered in the survivors compared with deceased patients [56.7 (43.3–116.7) ml/day/month (*p* < 0.001)]. A lower proportion of encephalopathy (37.1 vs 56.1%, *p* < 0.001), hypervolemia (24.5 vs 56.1%, *p* < 0.001) and higher proportion of AVF (30.8 vs 2.8%, *p* < 0.001), Diuretic (65.7 vs 36.4, *p* < 0.001), ACEI (67.8 vs 38.3, *p* < 0.001), ßeta blocker (21.0 vs 10.3, *p* < 0.001), EPO (64.3 vs 38.3, *p* < 0.001) were also observed in survivors patients in comparison to those who have died.

**Table 2:** Clinical and biological characteristics of deceased and survivors patients

Variables	All Group (*N* = 250)	deceased (*N* = 107)	survivor (*N* = 143)	*p*
Weight (Kg)*, *n* = 240	69.9 ± 13.9	67.3 ± 14.2	71.8 ± 13.5	0.012
BMI (Kg/m^2^)*, *n* = 130	24.9 ± 4.8	24.5 ± 4.2	25.1 ± 5.1	0.485
SBP, mm Hg, *n* = 250	153 ± 27.2	154 ± 26.4	153 ± 27.9	0.967
DBP, mm Hg, *n* = 250	84.7 ± 18.3	86 ± 17.4	83.6 ± 18.9	0.311
PP, mm Hg, *n* = 250	69.1 ± 20.7	67 ± 19.4	70 ± 21.6	0.231
Primary renal disease, *n* (%)				0.509
Glomerulonephritis	74 (29.5)	35 (32.7)	39 (27.3)	
Diabetic nephropathy	79 (31.6)	34 (31.8)	45 (31.5)	
Hypertensive nephropathy	64 (25.6)	24 (22.4)	40 (27.9)	
HIVAN	10 (4)	4 (3.7)	6 (4.2)	
All other	23 (9.2)	10 (9.3)	13 (9.1)	
IUV (ml/day)*, *n* = 250	550 (500–705)	250 (200–400)	840 (750–1000)	<0.001
RUV 3 months (ml/day)*, *n* = 200	500 (450–550)	260 (199–330)	700 (580–783)	< 0.001
RUV 6 months (ml/day)*, *n* = 127	500 (400–550)	250 (150–350)	560 (500–750)	< 0.001
RUV 12 months (ml/day)*, *n* = 77	400 (200–530)	200 (0–300)	500 (290–750)	0.003
Decline RUV (ml/day/month)*, *n* = 248	25 (20.8–33.3)	56.7 (43.3–116.7)	12.9 (8.3–16.7)	<0.001
Uremic encephalopathy, *n* (%)	113 (45.2)	60 (56.1)	53 (37.1)	0.001
Hypervolemia, *n* (%)	95 (38)	60 (56.1)	35 (24.5)	< 0.001
Diuretic, *n* (%)	133 (53.2)	39 (36.4)	94 (65.7)	< 0.001
ACE inhibitor, *n* (%)	138 (55.2)	41 (38.3)	97 (67.8)	< 0.001
Beta blocker, *n* (%)	41 (16.4)	11 (10.3)	30 (21.0)	0.011
EPO, *n* (%)	129 (51.6)	37 (34.6)	92 (64.3)	< 0.001
Vascular access, *n* (%)				< 0.001
catheter	203 (81.2)	104 (97.2)	99 (69.2)	
AVF	47 (18.8)	3 (2.8)	44 (30.8)	
Weekly hours of HD, *n* (%)				0.036
≤ 8	134 (53.6)	63 (58.9)	71 (49.7)	
≥ 12	116 (46.4)	44 (41.1)	72 (50.3)	
Weekly hours of HD*	8 (8–12)	8 (8–12)	12 (8–12)	0.034
Kt/V urea*, *n* = 133	1.3 ± 0.2	1.2 ± 0.2	1.3 ± 0.2	0.249
Charlson index*, *n* = 250	3.8 ± 2.6	4.3 ± 3.1	3.3 ± 1.9	0.003
Serum creatinine* (mg/dl), *n* = 240	16.3 ± 12.6	16.3 ± 10.2	12.6 ± 6.5	0.001
Urea *(mg/dl), *n* = 239	227.8 ± 110.7	259.9 ± 114.6	205 ± 102.2	< 0.001
Kaliemia*(mEq/l), *n* = 239	5.1 ± 1.4	5.4 ± 1.5	4.9 ± 1.2	0.001
Serum calcium* (mEq/l), *n* = 205	4.4 ± 0.7	4.3 ± 0.8	4.5 ± 0.6	0.119
Serum phosphate* (mg/dl), *n* = 91	4.9 ± 2.1	5.7 ± 2.1	4.7 ± 2.1	0.074
Serum albumine* (g/l), *n* = 215	37.6 ± 8.1	34.6 ± 8.5	40 ± 6.9	< 0.001
Hemoglobin* (g/dl), *n* = 249	8.5 ± 2.2	7.6 ± 1.9	9.2 ± 2.1	< 0.001
Hematocrit* (%), *n* = 247	25.8 ± 6.5	23.4 ± 5.9	27.5 ± 6.4	< 0.001
CRP* (mg/l), *n* = 86	14.3 ± 20.5	34 ± 31.7	9,1 ± 12.2	< 0.001

Clinical and laboratory data were collected firstly at the initiation of HD, excluding RUV at 3, 6 and 12 months after HD. Data are expressed as numbers and proportions in parentheses or mean ± standard deviation, median (interquartile range) as appropriate

Abbreviations: *BMI*: body mass index, *SBP*: systolic blood pressure, *DBP*: diastolic blood pressure, *PP*: pulse pressure, *HIVAN*: human Immunodeficiency virus associated nephropathy, *IRUV*: initial residual urine volume, *RUV*: residual urine volume, *ACE*: angiotensin conversion enzyme inhibitor, *EPO*: erythropoietin, *AVF*: arterio-venous fistula, *Kt/V urea*: clearance of urea ml/min based on the volume of distribution, HD: hemodialysis, CRP: C reactive protein

Furthermore, the survivors had longer median HD duration (12 h vs 8 h, *p* = 0034), higher serum albumin (40 ± 6.9 vs 34.6 ± 8.5, *p* < 0.001), hemoglobin (9.2 ± 2.1 vs 7.6 ± 1.9, *p* < 0.001), and lower serum level of urea (205 ± 102 vs 259.9 ± 114.6, *p* < 0.001), creatinine (12.6 ± 6.5 vs 16.3 ± 10.2, *p* = 0.001), potassium (4.9 ± 1.2 vs 7.6 ± 1.9, *p* = 0.001), CRP (9.1 ± 12.2 vs 34 ± 31.7, *p* ˂ 0.001) and lower Charlson index (3.3 ± 1.9 vs 4.3 ± 3.1, *p* = 0.003) than the group of deceased patients.

During the follow-up period, 143 (57.2%) patients survived, 17 (6.8%) were transplanted and 123 (49.2%) others still continue on maintenance HD. Three patients (1.2%) were lost to follow-up. One hundred and seven patients (42.8%) died, representing a mortality of 78 per 1000 patient-years, with a significant difference between persons who had preserved RUV (17 per 1000 patient-years) versus lost RUV (61 per 1000 patient-years). The first quarter of HD duration was the critical time to death. The main causes of death were the following: withdrawal from dialysis due to financial constraints (42.9%), infectious complications (17.7%), cardiovascular complications (15.8%), hyperkalemia (9.3%), neoplasia (9.3%), hemodynamic instability (1.8%) and nutritional depletion (0.9%). Table 3 compares the characteristics of deceased patients according to withdrawal from dialysis status. Between the both groups, there were significant differences in SEL, financing, EPO and weekly hours of HD. The withdrawal group had no patient with a high SEL (0 vs 13.1%, *p* = 0.005), unsecured financing (93.5 vs 67.2%, *p* = 0.001), not EPO (80.4 vs 52.5%, *p* =0.002) and had lower weekly hours of HD (69.6 vs 49.2%, *p* = 0.021) than other deceased.

**Table 3:** Characteristics of patients who died by withdrawal from dialysis of financial constraints compared to patients who died from other causes

Variables	Patients died by withdrawal from HD (*n* = 46)	Other causes of mortality (*n* = 61)	*p*
Gender, *n* (%)			0.093
Male	33 (71.7)	38 (62.3)	
Female	13 (28.3)	23 (37.7)	
Mean Age, years	50.7 ± 13.3	53.7 ± 13.1	0.260
Profession, *n* (%)			0.261
Unemployed	25 (54.3)	26 (42.6)	
Employed	21 (45.7)	35 (57.4)	
SEL, *n* (%)			0.005
Low	16 (34.8)	10 (16.4)	
Medium	30 (65.2)	43 (70.5)	
High	-	8 (13.1)	
Financing, *n* (%)			0.001
Not secured	43 (93.5)	41 (67.2)	
secured	3 (6.5)	20 (32.8)	
EPO			0.002
No	37 (80.4)	32 (52.5)	
Yes	9 (19.6)	29 (47.5)	
Vascular access, *n* (%)			
catheter	46 (100)	58 (95.1)	0.177
AVF	-	3 (4.9)	
IRUV* (ml/day)*, *n* = 250	240 (150–400)	290 (200–400)	0.939
Decline RUV* (ml/day/month), *n* = 248	66.7 (33.3–150)	50.8 (33.3–116.7)	0.693
Weekly hours of HD, *n* (%)			0.021
≤8	32 (69,6)	30 (49.2)	
≥12	14 (30,4)	31 (50.8)	
Duration in HD* (month)	4 (3–5)	4,5 (4–6)	0.180

Data are expressed as numbers and proportions in parentheses or mean ± standard deviation, median (interquartile range) as appropriate

Abbreviations: *SEL*: socio-economic level, *HD*: hemodialysis, *EPO*: erythropoietin, *IRUV*: initial residual urine volume, *RUV*: residual urine volume

*,quantitative variables

**Fig. 1:**
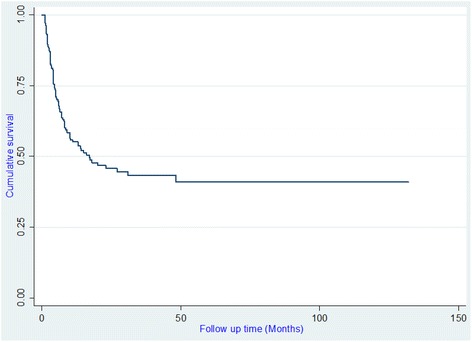
Survival Curves global of chronic hemodialysis patients

The Kaplan-Meier survival curve of initial HD can be viewed in Fig. 1. The median survival of the entire group was 17 months (Fig. 1). The cumulative survival rate of HD patients in this cohort study was 84.8% at 3 months, 71.6% at 6 months, 62.8% at one year, 58.4% at two years, 57.6% at three years and 57.2% at four years.

**Fig. 2:**
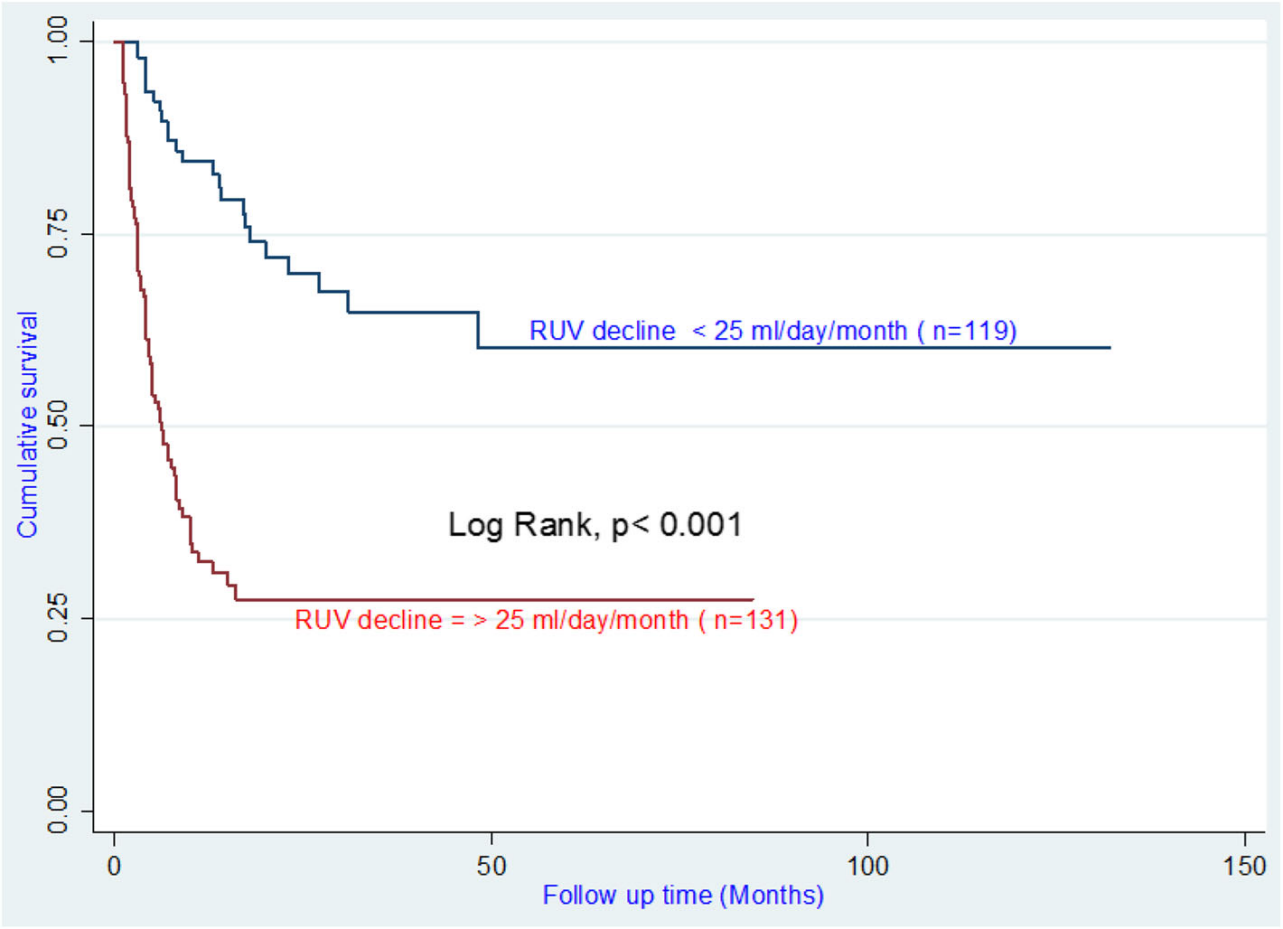
Survival Curves of chronic hemodialysis patients based on the decline residual urine volume

The Kaplan-Meier survival curves for preserved RUV and lost RUV are shown in Fig. 2. For the preserved RUV group, median follow-up time alive on dialysis was 30 months, and for lost RUV group 10 months. The differences in survival between preserved and lost RUV HD patients were significant (Log-rank: *p* < 0.001).

Based on a multivariate Cox regression analysis performed to estimate the risk factors for all-cause mortality in HD patients, adjusted survival rates of high decline versus low decline HD patients was significantly different (adjusted hazard ratio [aHR] 5.35, 95% CI [2.73–10.51], *p* < 0.001) (Table 4). Compared to patients having preserved RUV, the loss of RUV group increased nearly 5 times the risk of mortality. There were several others factors independently associated with mortality. Patients without secured financing had higher mortality rate (aHR 2.23, [1.11–4.46], *p* = 0.024) than those who had secured financing patients. Furthermore, patients with hypervolemia (aHR 2.00,95% CI [1.17–3.40], *p* = 0.011), catheter vs AVF (aHR 6.26, 95% CI [1.71–22.85], *p* = 0.006), lower serum albumin (aHR 0.93, 95% CI [0.89–0.96], *p* < 0.001) hemoglobin (aHR 0.73, 95% CI [0.63–0.84], *p* < 0.001) and lack of oral medication such as ACEI (aHR 2.48, 95% CI [1.40–4.40], *p* = 0.002) and Beta blocker (aHR 4.04, 95% CI [1.42–11.54], *p* = 0.009) had a significantly higher mortality.

**Table 4:** Mortality Predictors of hemodialysis patients in Kinshasa, with Cox regression

Variables	Univariate Analysis				Multivariate Analysis			
	HR	95,0%CI		*p*	Adjusted HR	95,0%CI		*p*
Financing (not secured vs yes)	3.33	2.16	5.15	<0.001	2.23	1.11	4.46	0.024
Weight *(Kg)	0.98	0.96	0.99	0.005	1.02	0.99	1.04	0.179
Decline RUV (≥25 vs <25 ml/day/month)	4.56	2.87	7.23	<0.001	5.35	2.73	10.51	<0.001
Hypervolemia (yes vs no)	2.79	1.90	4.09	<0.001	2.00	1.17	3.40	0.011
ACE inhibitor	2.53	1.71	3.74	<0.001	2.48	1.40	4.40	0.002
Beta blocker	2.56	1.37	4.79	0.003	4.04	1.42	11.54	0.009
Catheter vs AVF	18.84	5.89	60.26	<0.001	6.26	1.71	22.85	0.006
Weekly hours of HD ≤8 vs ≥12	1.56	1.06	2.31	0.024	1.70	0.91	3.17	0.095
Charlson Index*	1.08	1.01	1.15	0.022	1.01	0.94	1.09	0.812
Kaliemia*, mEq/l	1.17	1.02	1.35	0.027	1.12	0.92	1.35	0.272
Serum albumine*, g/l	0.94	0.92	0.97	<0.001	0.93	0.89	0.96	<0.001
Hemoglobin*, g/dl	0.731	0.66	0.81	<0.001	0.73	0.63	0.84	<0.001

Abbreviations: *RUV*: residual urine volume, *ACE*: angiotensin conversion enzyme inhibitor, *AVF*: arterio-venous fistula

*,quantitative variable

## Discussion

This work evaluated the benefit of RUV on the survival of chronic hemodialysis patients in Kinshasa, a large city in Sub-saharan Africa.

In this present cohort study, we found a mortality rate of HD patients of 78 per 1000 patient-years. The median survival was 17 months and the survival over the time was poor during the first two years compared to that yielded in developed countries before it stabilized at the third year or more, at the same level or above the survival level in the West [1, 3, 4, 21]. Lack of healthcare financing could explain the poor survival during the first two years. As expected, treatment discontinuation due to financial constraints was the primary cause of mortality in chronic HD Congolese patients. Only patients having secured financing HD continue the treatment beyond. Indeed, the financial situation in the DRC in 2013 showed that 80% of the urban population was poorwith a per capita GNP barely above 100 USD [22]. Considering that a single dialysis session costs at least 250 USD and that patients or families must pay all this cost in the absence of government funding or health insurance to cover the high costs of such treatment, it becomes clear that long-term dialysis will not an option for most Congolese with ESRD.. The cost estimates do not include the cost of drugs like erythropoietin, iron therapy, vitamin D analogues, antihypertensive etc., now considered an essential part of CKD management. These drugs raise the RRT costs by over 100% and are the exclusive preserve of the rich. Another prominent missing factor is the cost of AVFmaking, patient hospitalizations as well the cost of transport to and from HD center. Ultimately, the situation becomes untenable and the patient eventually stop dialysis and inexorably progress to death. Furthermore, the aging of patients in the West and in Japan combined with the presence of several comorbidities could explain the low survival encountered 5 years after initiation of dialysis in these countries. One study showed that mortality was 45 times lower among African Americans than Caucasians due to -among other reasons- the young age of African American patients [23].

This study showed that patients with preserved RUV had better survival in HD. These results corroborate those of several studies worldwide that have shown that the conservation of RUV is a better survival factor in HD [6–8, 10, 24–31]. Several mechanisms underlie the benefit of preserved RUV on survival. These include increased clearance of molecules of medium molecular weight, effective elimination of uremic toxins, maintenance of normovolemia, better control of blood pressure, prevention or regression of LVH, better control of phosphorous and calcium metabolism, decrease of malnutrition and inflammation and improvement of anemia [26].

The loss of residual urine volume, financing not secured of HD, hypervolemia, not taking ACE inhibitor and betablocker, central line catheter as a vascular access normal serum albumin levels and anemia have emerged as the main independent predictors of all-cause mortality in this study. This observation corroborates that reported by other studies that have found a link between these risk factors and mortality in chronic HD patients [30, 32–38].

In this study, a fast RVU decline retained in the dialysis fell almost 5 times the risk of death. The RRF was chosen as independent factor of mortality in The Netherlands Cooperative Study on the Adequacy of Dialysis (NECOSAD) -a prospective study conducted in the Netherlands [33], in a longitudinal cohort study of 6538 patients by Obi et al. in USA [5] and in other study conducted in China by Lo et al. [32].

Similarly, the hypervolemia is associated with high mortality in HD patients. Indeed, there is a close relationship between hypervolemia, pulse pressure, hypertrophy of left ventricle and fibroblast growth factor-23 (FGF-23) that are factors of morbidity and cardiovascular mortality in HD by contributing to atherosclerosis and vascular calcifications. It is noticeable that both hypervolemia and serum level of FGF-23 increase in case of the loss of RUV [11, 34–38].

Wu et al. examining 133,564 dialysis patients aged ≥18 years old concluded that overall mortality was significantly lower in dialysis patients who used an ACE inhibitor [39]. This is corroborated by the present study.

The benefit of beta-blocker in the survival of HD patients is demonstrated by a large retrospective study involving 50,468 US hemodialysis who concluded that only the use of beta-blocker increases from one year patient survival compared with ACE inhibitor and ARBs and calcium channel blockers [40]. In the present study, the use of beta blocker decreased by almost 4 times the risk of death.

This study found that patients with catheter had six times more likely to die than those with an arteriovenous fistula. It was reported that the catheter contributed to mortality in HD through not only infectious complications but also the associated cardiovascular risk especially in the first 3 months after initiation of HD [41].

It is established that hypoalbuminemia predicts mortality of dialysis patients and results from malnutrition of whomit is the ultimate biological expression. Its detrimental role in survival in HD is supported by numerous studies [42, 43].

In the present cohort, the mean Hb was low at 8.5 g/dl at admission and Hb level was an independent predictor of mortality in HD. Jung et al. in a multicenter prospective observational study report that a Hb level < 9 g/dl multiply by 4 the all-cause mortality in HD [44].

The unsecured financing HD was found to be independent predictors of mortality and each multiplied by 2 the risk of death. This factor contribute to high mortality the first months of HD observed in this study where 46 patients died by withdrawal from dialysis due to financial constraints. The discontinuation of treatment because of financial constraints which raises the thorny issue of healthcare financing faced by almost all countries in Sub-Saharan Africa and calls for more responsibility from political and health authorities to reform the health system [45, 46].

The interpretation of the results of this study should consider some limitations. Its retrospective character has not allowed to obtain all data related to the parameters of interest. The accuracy of the 24-h urine collection could not be established due to lack of a measured value for urinary creatinine. The use of residual urine volume, not direct measures including renal urea clearance and/or creatinine clearance, is another limitation. The information related to the reference time are indeed very imprecise. However, there were no statistically significant difference between the survivor group and the deceased in relation to these parameters, but it is known that the late reference to the nephrologist is a poor prognostic factor for survival in dialysis. There is also a real selection bias. Patients who can afford hemodialysis belong either to a medium or high socioeconomic level, which is far from the majority of patients with ESRD whose social level economic is rather low and does not even have the ability to attend Nephrology center. Moreover, this study has a specific distribution of cause of death; the most frequent cause of death was withdrawal from dialysis because of financial constraints, which is rarely observed in developed countries. Therefore, the results of this study cannot be extrapolated to the other areas where health insurance well covers dialysis treatment. Nevertheless, this study has the advantage of being the first one in the country to determine the impact of the preserved RUV on the survival in HD even in a context of bad financial position.

## Conclusion

The median survival of hemodialysis patients in Kinshasa was relatively high in case of preservation of the RUV. The loss of the RUV in HD was an independent predictor of all-cause of mortality, in parallel with the unsecured financing HD, hypervolemia, anemia, absence of ACE inhibitor or betablocker, presence of catheter as a vascular access and hypoalbuminemia. This is a good reason to elucidate the predictors of preserving RUV for better support for HD patients in our communities.

## Abbreviations

ACE: Angiotensin conversion enzyme
AVF: Arterio-venous fistula
BMI: Body mass index
CI: Confidence interval
CRP: C reactive protein
EPO: Erythropoietin
ESRD: End-stage chronic renal disease
HD: Hemodialysis
HR: Hazard ratio
IRUV: Initial residual urine volume
Kt/V urea: Clearance of urea ml/min based on the volume of distribution
LVH: Left ventricular hypertrophy
P: Statistical significance
RRF: Residual renal function
RUV: Residual urine volume
SEL: Socio-economic level
